# Pharmacovigilance of triazole antifungal agents: Analysis of the FDA adverse event reporting system (FAERS) database

**DOI:** 10.3389/fphar.2022.1039867

**Published:** 2022-12-15

**Authors:** Jianxing Zhou, Zipeng Wei, Baohua Xu, Maobai Liu, Ruichao Xu, Xuemei Wu

**Affiliations:** ^1^ Department of Pharmacy, Fujian Medical University Union Hospital, Fuzhou, Fujian, China; ^2^ School of Pharmacy, Fujian Medical University, Fuzhou, Fujian, China; ^3^ Department of Pharmaceutical Sciences, School of Pharmacy, University of Pittsburgh, Pittsburgh, PA, United States

**Keywords:** triazole antifungal agents, pharmacovigilance, adverse events, information component, spectrum, isavuconazole (DRUG)

## Abstract

Triazole antifungal drugs (TAD) are widely used to treat invasive fungal infections due to their broad antifungal spectrum and low toxicity. Despite their preference in the clinic, multiple Adverse Events (AE) are still reported each year. Objective: We aimed to characterize the distribution of Adverse Events associated with Triazole antifungal drugs in different systems and to identify Important Medical Events (IME) signals for Triazole antifungal drugs. Methods: The U.S. Food and Drug Administration Adverse Event Reporting System (FAERS) was queried for Adverse Events related to Triazole antifungal drugs from 2012 to 2022. The Adverse Events caused by all other drugs and non-TAD antifungal drugs were analyzed as references. Reporting odds ratio and Bayesian confidence propagation neural network of information components were used to evaluate the association between Triazole antifungal drugs and Important Medical Events. Visual signal spectrum is mapped to identify potential adverse reaction signals. Results: Overall, 10,262 Adverse Events were reported to be associated with Triazole antifungal drugs, of which 5,563 cases were defined as Important Medical Events. Common adverse drug reactions (ADR) mentioned in the instructions such as delirium and hypokalemia were detected, as well as unlabeled ADRs such as rhabdomyolysis and hepatitis fulminant. Cholestasis, drug-induced liver injury, QT interval prolongation and renal impairment have notable signals in all Triazole antifungal drugs, with 50 percent of patients developing a severe clinical outcome. Isavuconazole had the lowest signal intensity and demonstrated a superior safety profile. Conclusion: Most results are generally consistent with previous studies and are documented in the prescribing instructions, but some IMEs are not included, such as hepatitis fulminant. Additional pharmaco-epidemiological or experimental studies are required to validate the small number of unlabeled ADRs. TAD-related Important Medical Eventshave a considerable potential to cause clinically serious outcomes. Clinical use of Triazole antifungal drugs requires more attention.

## 1 Introduction

Antifungal drugs including triazole, polyene, flucytosine and echinocandins are commonly used for the treatment of invasive fungal infections caused by *Candida* spp, Cryptococcus spp. and Aspergillus spp. (Pappas et al., 2016). The major advantage of triazole antifungal drugs (TAD) over amphotericin B is their broader antifungal spectrum, less toxicity, and better tolerability, which made them become the most prescribed anti-fungal drugs in clinical practice (Gomes et al., 2014). Ergosterol biosynthesis pathway is the target of TAD. By inhibiting the cytochrome P450-dependent enzyme sterol 14α-demethylase, TAD can lead to the accumulation of 14α-methylated sterols in the fungal cell membrane, the disruption of the structure and function of cell membranes, and the change in cell permeability, ultimately resulting in cell death (Sun et al., 2007; Gubbins, 2011). Commercially available TAD include first-generation products fluconazole and itraconazole, and second-generation products voriconazole, posaconazole and isavuconazole. Common ADRs include rash, headache, gastrointestinal reactions, QT abnormalities, and liver injury, which could potentially lead to liver failure in rare cases. Although research (Maertens et al., 2016) has shown that the toxicity of the new generation of TADs has decreased compared to their predecessors, there has been an upward trend in reports of adverse reactions (ADRs) with the widespread use of TADs in recent years.

The U.S. Food and Drug Administration (FDA) Adverse Event Reporting System (FAERS) is a database designed to support the FDA’s post-marketing surveillance for drugs and therapeutic biologicals. The database includes all the adverse events (AEs) and medication errors collected by the FDA, which allows for signal detection and quantitative analysis of the reported drug-related AEs (Sakaeda et al., 2013). The reporting information structure of the FAERS database adheres to the International Safety Reporting Guidelines (ICH E2B) issued by The International Council for Harmonization of Technical Requirements for Pharmaceuticals for Human Use (ICH), and AE and medication error in the reports are coded according to the ICH International Dictionary of Medical Terms (HHS Food and Drug Administration, 2016).

Important medical events (IME) are defined by the European Medicines Agency (EMA), with the aim of prioritizing the review of the serious ADRs among AEs. IMEs are also used to identify the suspected ADRs of concern (Vogel et al., 2020). Comprehensive analysis of IMEs helps to identify the serious TAD-triggered AEs that are not included in the drug labels, to reveal all the potential adverse reaction signals of TAD. Therefore, mapping the IME signal spectrum is of great importance for clinical medication safety.

This study aims to analyze all the TAD-related IMEs at the system organ classification (SOC) level by mining the adverse reaction database FAERS and plotting the signal spectrum. Visual graphs showing the correlation between TAD and IMEs enable fast identification of potential safety issues and provide recommendations for clinical use.

## 2 Methods

### 2.1 Data sources and processing methods

FAERS data are released quarterly. Each FAERS data contains seven different files: Patient Demographic Information Form (DEMO), Drug/Biological Information Form (DRUG), Adverse Reaction Form (REAC), Clinical Outcome Form (OUTC), Reporting Source Form (RPSR), Drug Therapy Start and End Date Form (THER), and Use/Diagnosis Indication Form (INDI). Structured Query Language (SQL) was utilized to extract and analyze all the reported AEs of target TAD (fluconazole, itraconazole, voriconazole, posaconazole, and isavuconazole) in the FAERS database from the fourth quarter of 2012 to the first quarter of 2022. Considering the credibility of the results, we analyzed the reports focusing on the most suspected drugs that were deemed to cause the adverse events with a role_cod of PS (drug role as the primary suspect role). According to FDA recommendations, the following criteria was used to screen out the duplicate data: when case_id and fda_dt were the same, duplicate records under the same case were removed while keeping the latest fda_dt (Hu et al., 2020). Records with flaws or mixed informative quality, such as age>150, cases having same ID but different sex or earlier than 2012, were deleted.

**FIGURE 1:**
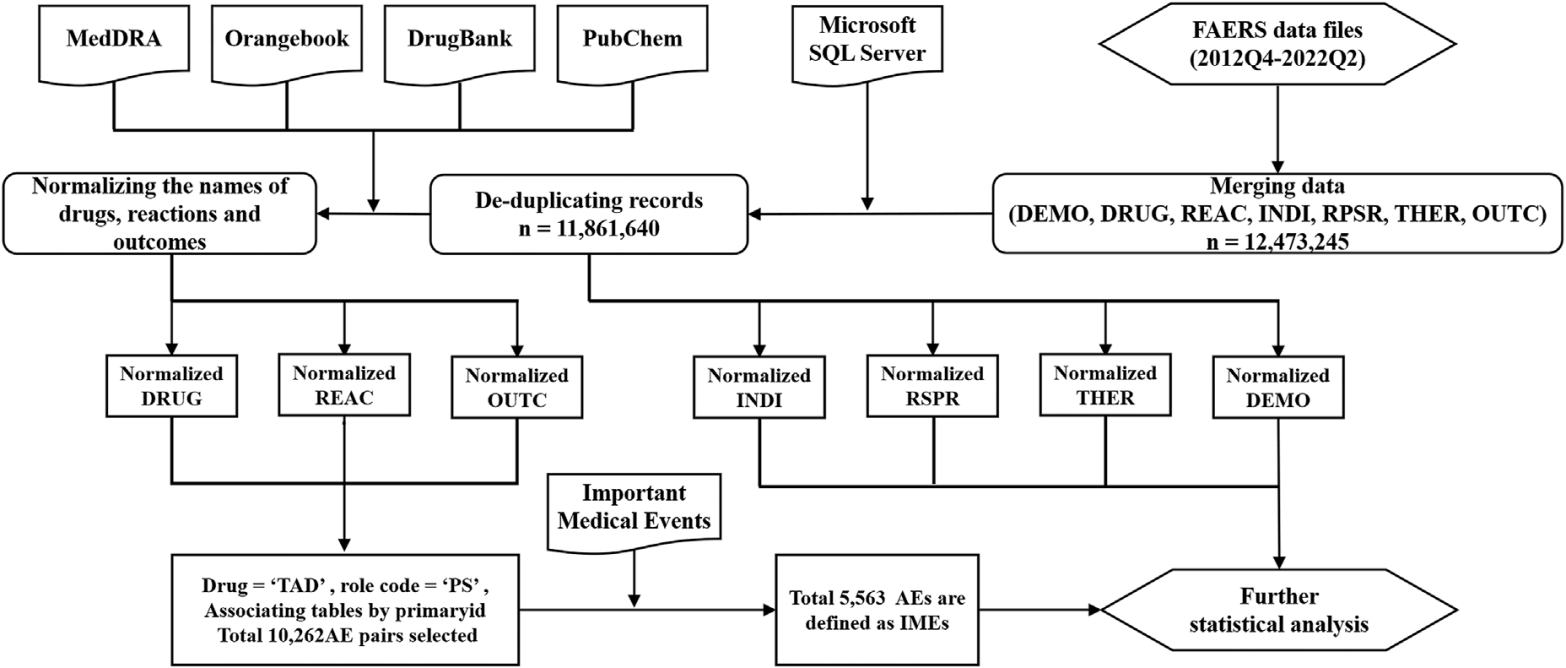
The main steps in the processing of the FAERS database (DEMO: demographics; DRUG: drug; REAC: reaction; INDI: indication; RPSR: reporting source; THER: therapy; OUTC: outcome. All seven tables above are from FAERS data file).

Due to the different disciplinary background of the reporters, the reported drug names and adverse reaction names in the FAERS database are not standardized and uniform. To identify records of target drugs in FAERS, we collected trade names, generic names and chemical names of target TAD in the FDA Orange Book, the National Institutes of Health Organic Small Molecule Bioactivity Database (PubChem) and the University of Alberta Bioinformatics and Cheminformatics Database (Drug Bank). All the names used to retrieve TADs are shown in Online Resource 1. All the AEs were coded as primary terms (PTs) according to the Medical Dictionary for Regulatory Activities version 25.0 (MedDRA 25.0). SOCs corresponding to these PTs were also listed. Serious clinical outcomes were defined as death, life-threatening, disability, and hospitalization. Unexpected AEs were defined as any AE not mentioned in the FDA drug prescription information. Figure 1presents the main steps.

### 2.2 Data analysis

Given that disproportionality emerges when a specific AE is associated with a given drug, two standard Disproportionality Analyses (DPA) methods, Reporting Odds Ratio (ROR) and Bayesian confidence propagation neural network of information components (IC), were applied to detect any potential positive signal (Rothman et al., 2004; Tada et al., 2020). Considering that a spurious association can occur when events with very low expected frequency, statistical shrinkage transformation was performed to obtain conservative results (Norén et al., 2013; Zhai et al., 2019). The shrinkage transformation for ROR and IC is as follows:

$$ROR=\frac{N_{observed}+0.5}{N_{expected}+0.5}$$

$$IC=\log_{2}\frac{N_{observed}+0.5}{N_{expected}+0.5}$$

$$N_{expected}=\frac{N_{drug}\times N_{event}}{N_{total}}$$

$${ROR}_{025}=e^{\ln ROR-1.96\times \sqrt{\frac{1}{a}+\frac{1}{b}+\frac{1}{c}+\frac{1}{d}}}$$

$${IC}_{025}=IC-3.3\times (N_{observed}+0.5^{-0.5}-2\times (N_{observed}+0.5^{-1.5}$$

All the DPA analyses are based on the same calculation principles using the 2 × 2 table (Table 1). Nobserved = a, which is the number of records observed for the drug-AE pair of interest; Nexpected is the expected number of records for the drug-AE pair of interest; Ndrug is the total number of records for the target drug; Nevent is the total number of records for the target AE; and Ntotal is the total number of records for the entire database.

**TABLE 1:** Two-by-two contingency table for the disproportionality analysis.

	Target AEs	Other AEs
Target drugs	a	b
Other drugs	c	d

AEs, adverse effects; a is the number of reports of interested drug-AE, pairs; b is the number of reports with all other AEs, of the target drugs; c is the number of reports with the target AEs, of all other drugs; d is the number of reports with all other AEs, of all other drugs; In FAERS, all AEs, are coded as PTs, according to MedDRA., We matched all PTs, with the corresponding primary SOCs, and subsequently performed disproportionality analysis at the level of SOCs. At the SOC, level, a is the number of reports of interested drug-SOC, pairs; b is the number of reports with all other SOCs, of the target drugs; c is the number of reports with the target SOCs, of all other drugs; d is the number of reports with all other SOCs, of all other drugs.

The 95% confidence intervals were calculated for ROR (ROR025) and IC (IC025). A significant signal was defined as ROR025 greater than 1 with at least 3 cases or IC025 above 0 (van Puijenbroek et al., 2002; Scholl and van Puijenbroek, 2016). All the analyses were performed using SAS version 9.4 (SAS Institute Inc, Cary, NC, United States). The mapping matching of AEs and IMEs was completed using SQL and the signal spectrum of TAD-related IMEs were plotted.

## 3 Results

### 3.1 Descriptive analysis

From the fourth quarter of 2012 to the first quarter of 2022, a total of 11,861,640 AEs were reported to FDA, of which 10,262 cases was taken TAD as the primary suspected drug, and 5,563 of these TAD-related AEs (54.21%) were defined as IMEs. Table 2 depicts the distribution of various TAD in AEs and IMEs. The percentage of AEs and IMEs was highest for voriconazole (37.00% and 42.65%), followed by posaconazole (21.32% and 20.96%), with the remaining TADs having similar percentages. The proportion of AEs and IMEs was very similar for all the TADs analyzed, except for voriconazole, which had a slightly higher proportion of IMEs than AEs (5.65% higher).

**TABLE 2:** Distribution of TAD in AEs and IMEs.

Drug name	TAD-related AEs n (%)	TAD-related IMEs n (%)
Total	10,262	5563
Fluconazole	1384 (13.49)	659 (11.84)
Itraconazole	1154 (11.25)	629 (11.30)
Voriconazole	3797 (37.00)	2372 (42.65)
Posaconazole	2188 (21.32)	1166 (20.96)
Isavuconazole	1739 (16.95)	737 (13.25)

Table 3 describes the clinical characteristics of the patients in AEs and IMEs. There was an outbreak of *Candida* auris infection around the world in 2017, more TAD-related AEs (14.10%) or IMEs (11.22%) might enter FAERS due to the increased concern of the U.S. medical authorities. The percentage of reports in 2013, 2016 were 10.09% and 11.05% for AEs, 11.23% and 11.45% for IMEs, respectively, while around 8–10% appeared in other years. We assumed it is in a normal range of fluctuations. Considering the proportion of case with the unknown or missing gender information, it seems that male had higher occurrence in AEs and IMEs than female (47.97% vs. 39.28% in AEs, and 51.14% vs. 36.40% in IMEs, respectively). The median age of AEs and IMEs was 60 years (interquartile 44–70 years) and 62 years (interquartile 45–71 years), respectively. For both AEs and IMEs, more than half of the reports with known age information were observed in the elderly aged 60 or over. There is an overall upward trend in the number of reported cases per year, which may be due to an increased public concern to the TAD-related AEs (Benitez and Carver, 2019). During the investigation period (approximately 10 years), 2,357 patients with serious medical outcomes (including Death, Disability, and Life-Threatening) were reported in IMEs, suggesting that TAD may have potentially dangerous attributes.

**TABLE 3:** Characteristics of patients with AEs and IMEs associated with TAD.

	TAD-related AEs n (%)	TAD-related IMEs n (%)
Total	10,262	5563
Gender		
Male	4923 (47.97)	2845 (51.14)
Female	4031 (39.28)	2025 (36.40)
Unknown or missing	1308 (12.75)	693 (12.46)
Age (year)^a^		
median (interquartile)	60 (44–70)	62 (45–71)
0–17	594 (5.78)	338 (6.09)
18–59	2791 (27.20)	1614 (29.01)
60–74	2560 (24.95)	1555 (27.95)
75–89	1077 (10.50)	714 (12.83)
≥90	47 (0.46)	36 (0.65)
Unknown or missing	3193 (31.11)	1306 (23.48)
Year		
2012 (Q4^b^)	323 (3.15)	212 (3.81)
2013	1035 (10.09)	625 (11.23)
2014	913 (8.90)	495 (8.90)
2015	944 (9.20)	508 (9.13)
2016	1050 (10.23)	550 (9.89)
2017	1447 (14.10)	624 (11.22)
2018	985 (9.60)	570 (10.25)
2019	1005 (9.79)	536 (9.64)
2020	809 (7.88)	468 (8.41)
2021	1134 (11.05)	637 (11.45)
2022 (Q1^c^)	617 (6.01)	338 (6.08)
Reporter Country		
United States	5559 (54.17)	2273 (40.86)
Japan	879 (8.57)	642 (11.54)
France	823 (8.02)	635 (11.41)
China	671 (6.54)	454 (8.16)
Other countries	1996 (19.45)	1370 (24.63)
Outcome^b^		
Death	2240 (21.83)	1920 (34.51)
Life-Threatening	365 (3.56)	296 (5.32)
Hospitalization	2791 (27.20)	1888 (33.94)
Disability	193 (1.88)	108 (1.94)
Congenital Anomaly	26 (0.25)	24 (0.43)
Required intervention	11 (0.11)	9 (0.16)
Other Serious	4440 (43.27)	3543 (63.69)

^a^ Patients were grouped according to the United Nations World Health Organization’s criteria for age classification: children under 18, young adults 18–59, pre-geriatric age 60–74, mid-geriatric age 74–89, and terminal age 90+.

^b^ Q1 indicates that only the first quarter data is included, as is Q4.

^c^ For Outcome, because patients may experience multiple outcomes in a single AE, event, the total sum of percentages will be greater than 100%, but this is not conflicting.

### 3.2 SOCs analysis

Table 4 presents the signal intensity of TAD-related AEs and IMEs. Significant signal overlap for all the TAD occurs in five SOCs: “Blood and lymphatic system disorders” (IC025 of AEs = 0.19, ROR025 of AEs = 1.16; IC025 of IMEs = 0.58, ROR025 of IMEs = 1.16), “Endocrine disorders” (1.24, 2.42; 1.22, 2.40), “Hepatobiliary disorders” (1.61, 3.08; 1.93, 3.85), “Immune system disorders” (0.51, 1.44; 0.03, 1.04), and “Infections and infestations” (0.61, 1.53; 0.92, 1.89). In addition, TAD-related AEs showed significant signals in SOCs such as “Eye disorders” (IC025 of AEs = 0.32, ROR025 of AEs = 1.26) and “Investigations” (0.26, 1.20). IMEs showed significant signals in SOCs such as “Cardiac disorders” (IC025 of IMEs = 0.32, ROR025 of IMEs = 1.26), “Neoplasms benign, malignant and unspecified” (0.16, 1.33) and “Renal and urinary disorders” (0.17, 1.14).

**TABLE 4:** Signal strength of TAD-related AEs/IMEs at the SOC level.

SOC	TAD-related AEs			TAD-related IMEs		
	N	IC025	ROR025	N	IC025	ROR025
Blood and lymphatic system disorders^a^	602	0.19	1.16	435	0.58	1.52
Cardiac disorders^c^	723	0.09	0.95	530	0.32	1.26
Congenital, familial and genetic disorders^b^	114	0.01	1.04	62	-0.10	0.98
Ear and labyrinth disorders	107	-0.62	0.67	23	-2.31	0.22
Endocrine disorders^a^	207	1.24	2.42	117	1.22	2.40
Eye disorders^b^	780	0.32	1.26	161	-1.21	0.44
Gastrointestinal disorders	1853	-0.53	0.70	266	-2.57	0.17
General disorders and administration site conditions	4954	-0.15	0.89	1006	-1.63	0.32
Hepatobiliary disorders^a^	806	1.61	3.08	556	1.93	3.85
Immune system disorders^a^	547	0.51	1.44	225	0.03	1.04
Infections and infestations^a^	2449	0.61	1.53	1668	0.92	1.89
Injury, poisoning and procedural complications	3146	-0.06	0.96	83	-4.72	0.04
Investigations^b^	2168	0.26	1.20	110	-3.40	0.10
Metabolism and nutrition disorders	626	-0.13	0.92	184	-1.13	0.47
Musculoskeletal and connective tissue disorders	680	-1.34	0.40	83	-3.72	0.08
Neoplasms benign, malignant and unspecified^c^	603	-0.61	0.66	561	0.16	1.13
Nervous system disorders	1609	-0.69	0.62	629	-1.21	0.44
Pregnancy, puerperium and perinatal conditions	37	-2.29	0.22	16	-2.89	0.15
Product issues	130	-2.18	0.23	1	---	---
Psychiatric disorders	1053	-0.76	0.60	420	-1.26	0.42
Renal and urinary disorders^c^	536	-0.30	0.82	410	0.17	1.14
Reproductive system and breast disorders	68	-2.38	0.20	7	-5.59	0.02
Respiratory, thoracic and mediastinal disorders	1298	-0.19	0.88	390	-1.12	0.47
Skin and subcutaneous tissue disorders	1486	-0.26	0.84	198	-2.43	0.19
Social circumstances	43	-2.06	0.25	4	-5.71	0.02
Surgical and medical procedures	227	-0.98	0.52	0	---	---
Vascular disorders	382	-0.85	0.56	127	-1.68	0.32

^a^ TAD-related AEs/IMEs, are matched with IC_025_ value >0 and ROR_025_ value >1.

^b^ TAD-related AEs, are matched with IC_025_ value >0 and ROR_025_ value >1, while IMEs, are not.

^c^ TAD-related IMEs, are matched with IC_025_ value >0 and ROR_025_ value >1, while AEs, are not.

The above values that match IC_025_ value >0 and ROR_025_ value >1 are marked in bolded.

### 3.3 IMEs analysis

#### 3.3.1 Signal spectrum based on all other drugs as analysis context

**FIGURE 2:**
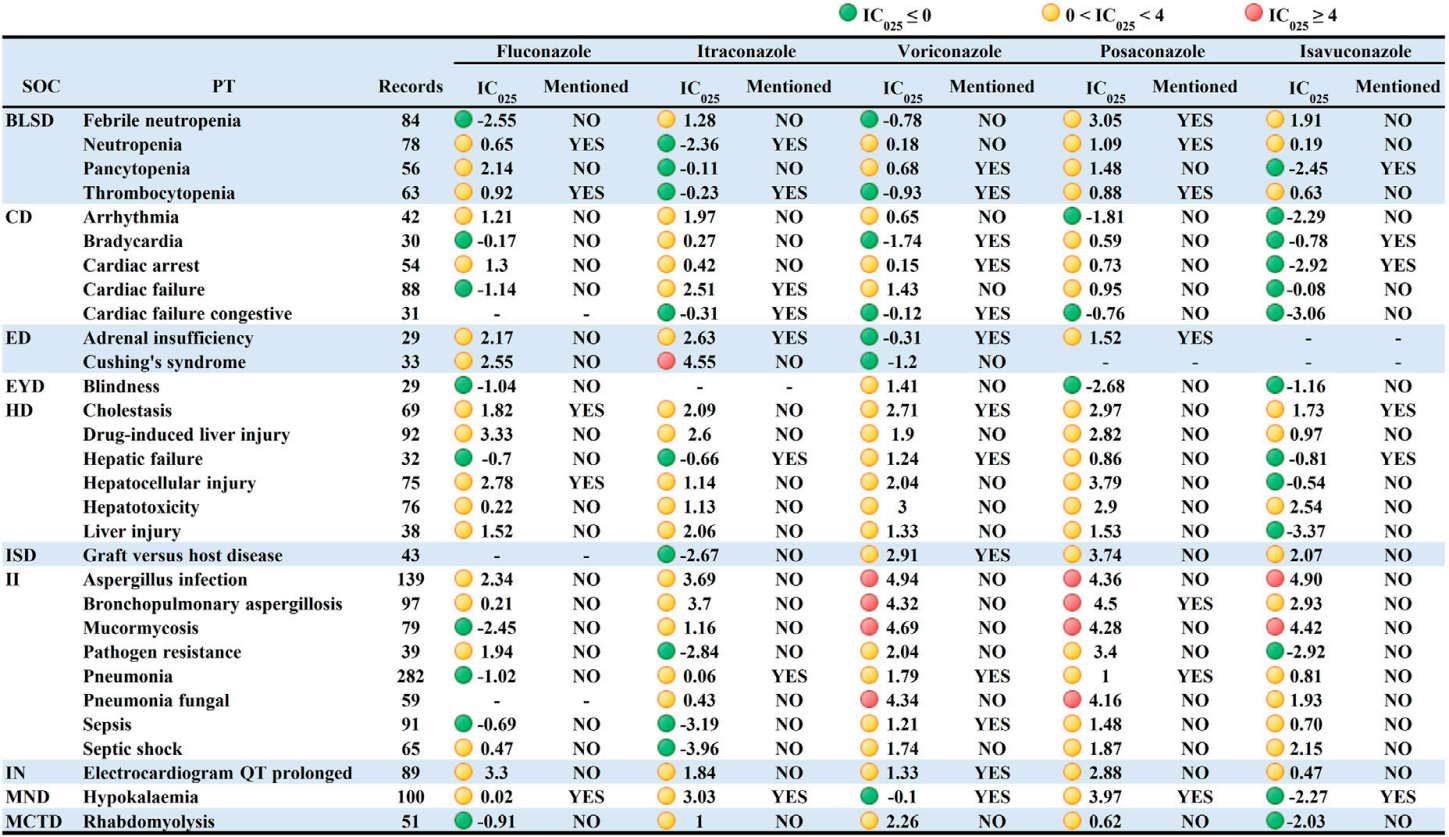
Signal profiles of top 30 IMEs induced by TAD based on all other drugs as analysis contexts. BLSD: Blood and lymphatic system disorders; CD: Cardiac disorders; ED: Endocrine disorders; EYD: Eye disorders; HD: Hepatobiliary disorders; ISD: Immune system disorders; II: Infections and infestations; IN: Investigations; MND: Metabolism and nutrition disorders; MCTD: Musculoskeletal and connective tissue disorders; IMEs: Important Medical Events; SOC: System Organ Class; PT: Preferred Term; IC_025_: the lower limit of the 95% confidence interval of IC. IC_025_ greater than 0 was deemed a signal. IC_025_ no less than four was deemed a strong signal.

Taken AEs caused by all other drugs in the FAERS database as a background, this study further analyzed the PT signal for each TAD in each specific IME to explore whether there was an association between them. Since specific IMEs less than 3 cases may result in false positive signals, they were not included in the analysis (Zhou et al., 2021). We used the IC025 value as an indicator and defined IC025 > 4 as a strong signal. The visualized signal spectrum was plotted, and the top 30 significant signals are listed in Figure 2 (421 in total, Online Resource two listed the full data). The PTs are listed in the corresponding SOCs in an ascending alphabetical order.

Signal spectrum indicated that TAD-related IMEs were mainly distributed in the category coded as “Infections and infestations” (n = 92, 21.85%) and “Nervous system disorders” (n = 42, 9.98%). Although fewer PTs were involved, TAD also showed strong signals in “Psychiatric disorders”, “Endocrine disorders " and “Neoplasms benign, malignant and unspecified”. This result agrees with the strength of the association between IMEs and specific SOCs in Table 4.

From a drug-specific perspective, voriconazole presented the broadest spectrum, with a total of 139 potential signals detected, ranging from *pseudomonas* infection (IC025 = 0.02) to leukemia (IC025 = 4.99). Besides the IMEs listed in the instructions (cholestasis, graft *versus* host disease, delirium, hallucinations, respiratory failure, and pseudoporphyria), 113 unlabeled IMEs were observed for voriconazole, with rhabdomyolysis, dermatitis bullous, inappropriate antidiuretic hormone secretion, and chloropsia showing stronger signal intensity. For posaconazole, a total of 96 potential signals were detected, ranging from immunodeficiency (IC025 = 0.05) to bronchopulmonary aspergillosis (IC025 = 4.50). Stronger signals were observed in bronchopulmonary aspergillosis, pneumonia fungal and mucormycosis. The latter two were not included in the instructions. Seventy-nine potential signals were detected for fluconazole, with torsade de pointes (IC025 = 3.57), QT interval prolonged (IC025 = 3.30), drug-induced liver injury (IC025 = 3.33), encephalitis (IC025 = 3.02) and erythema multiforme (IC025 = 3.35) showing significant correlations (The first three also showed signal intensity in other TAD). A total of 57 potential signals were detected for itraconazole. In addition to the seven known IMEs (e.g., cardiac failure, adrenal insufficiency, and hypokalaemia), 50 IMEs including Cushing’s syndrome were not mentioned in the instructions. Forty-five potential signals were detected for isavuconazole, with the characteristics of small in number, low in signal intensity and overlapped with other TAD. Notably, multiple organ dysfunction syndrome, cholestasis, liver injury, QT interval prolonged, aspergillus infection and renal impairment were the 6 PTs mentioned in the signal spectrum for all the TAD. The QT interval prolongation caused by TAD has been reported in the recent articles (Molloy et al., 2018; Yu and Liao, 2022), and the results of our study are consistent with it.

#### 3.3.2 Signal spectrum based on non-TAD antifungal drugs as analysis context

**FIGURE 3:**
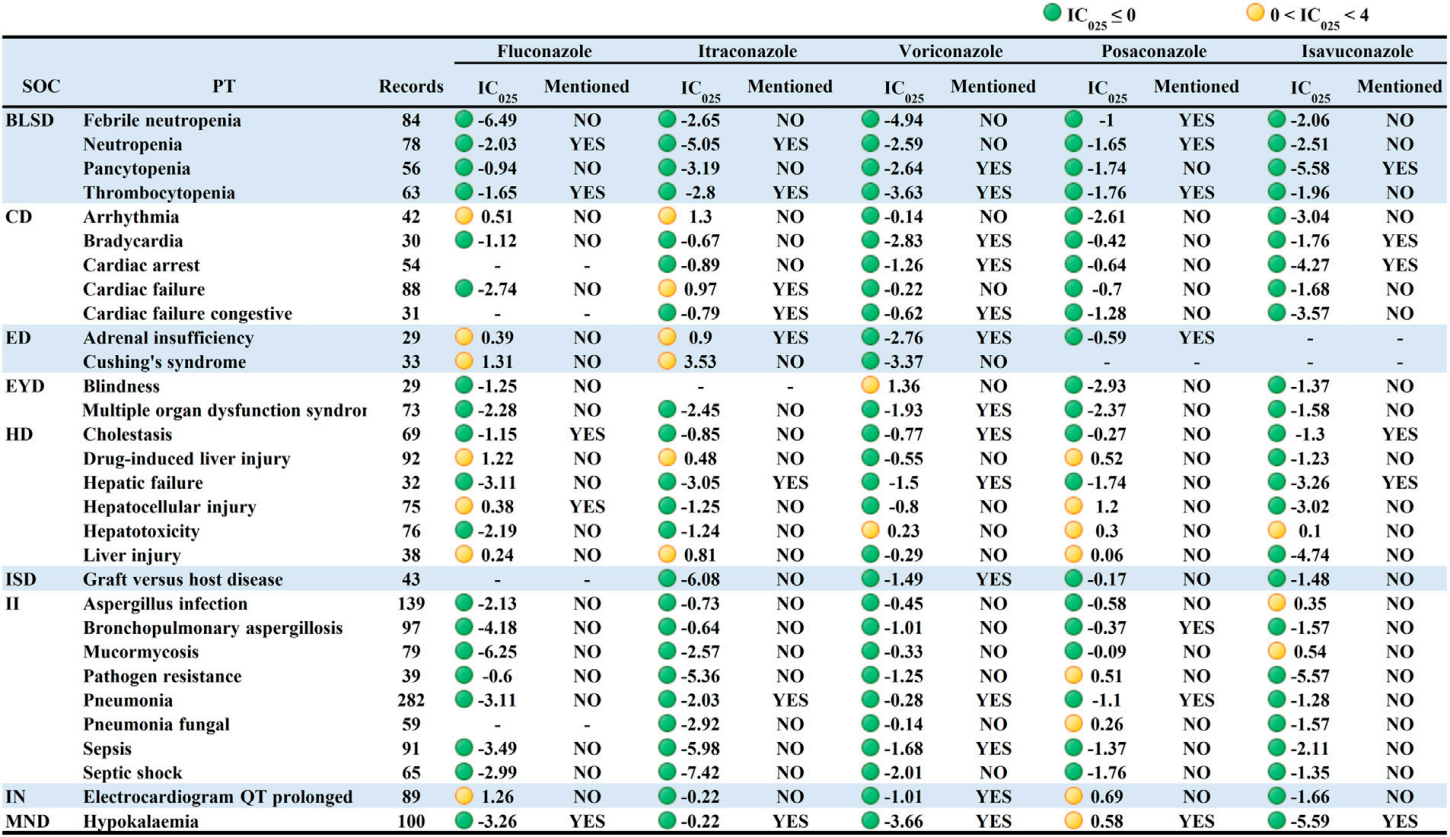
Signal profiles of top 30 IMEs induced by TAD based on non-TAD antifungal drugs as Analysis Contexts. BLSD: Blood and lymphatic system disorders; CD: Cardiac disorders; ED: Endocrine disorders; EYD: Eye disorders; HD: Hepatobiliary disorders; ISD: Immune system disorders; II: Infections and infestations; IN: Investigations; MND: Metabolism and nutrition disorders; IMEs: Important Medical Events; SOC: System Organ Class; PT: Preferred Term; IC_025_: the lower limit of the 95% confidence interval of IC. IC_025_ greater than 0 was deemed a signal. IC_025_ no less than four was deemed a strong signal.

Analysis shows that some of the PTs may be associated with the patients’ own potential diseases, which can cause some statistical bias (e.g., multiple strong positive signals were associated with fungal infections, which may be caused by the patients’ own disease rather than medication-induced fungal infections). To minimize the bias, AEs caused by non-TAD antifungal drugs were used as analysis context (The complete data can be viewed in Online Resource 3). Figure 3 showed an overall decrease in the IC025 values of IMEs and an absence of strong signals. The signals based on the analysis context of non-TAD antifungal drugs were mainly distributed in “Infections and infestations” and " Nervous system disorders”, which is similar to the results based on the analysis context of all other drugs.

Similar to the previous result, voriconazole had the broadest signal spectrum, with 38 potential signals detected. The strong IMEs for voriconazole found in Figure 2 were also observed but with considerably lower intensity. Toxic optic neuropathy, hallucinations, neoplasm malignant and pseudo porphyria had relatively strong signals (corresponding IC025 = 1.66, 2.44, 1.96, 1.99). The following was fluconazole (36 potential signals in total) with torsade de pointes, ventricular tachycardia, and erythema multiforme having the most significant signals (1.96, 1.64, 2.51). Posaconazole (with 21 potential signals) had the strongest signals for hepatocellular injury, leukemia, and QT interval prolongation (1.2,1.44,0.69). Itraconazole (17 potential signals) had relatively high signal levels (3.53, 1.37, 1.37) for Cushing’s syndrome, hypoplastic left heart syndrome, and arrhythmia. Isavuconazole had only eight potential signals with weakly positive intensity.

#### 3.3.3 Multidrug combination analysis

**FIGURE 4:**
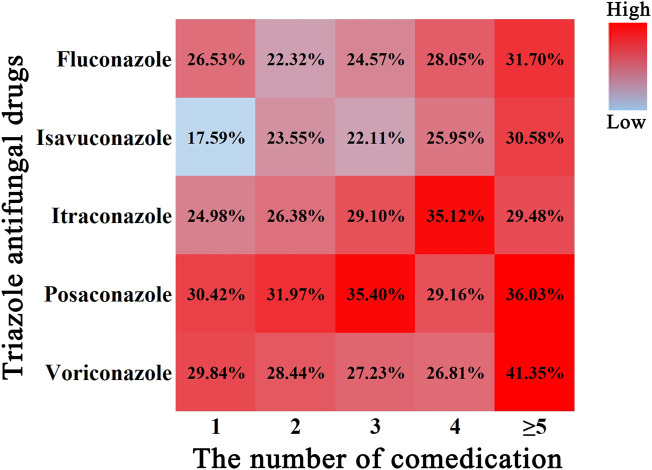
Color scale plot of the association of TAD-related IME with the number of comedication. Red color blocks indicate a high probability of IME, blue color blocks indicate a low probability of IME, and the percentages are the corresponding IME incidence.

A color scale was plotted to depict the relationship between the incidence of IME and multidrug combinations (Figure 4.) In general, the rise in incidence of IME was paralleled by an increase in the number of comedication. Isavuconazole alone had the lowest incident of IME (17.59%), while voriconazole administrated with more than five drugs had the highest value (41.35%). Posaconazole showed a relatively even and higher harmfulness, while isavuconazole and fluconazole displayed superior safety (color block closer to blue).

#### 3.3.4 Analysis of significant IMEs

**FIGURE 5:**
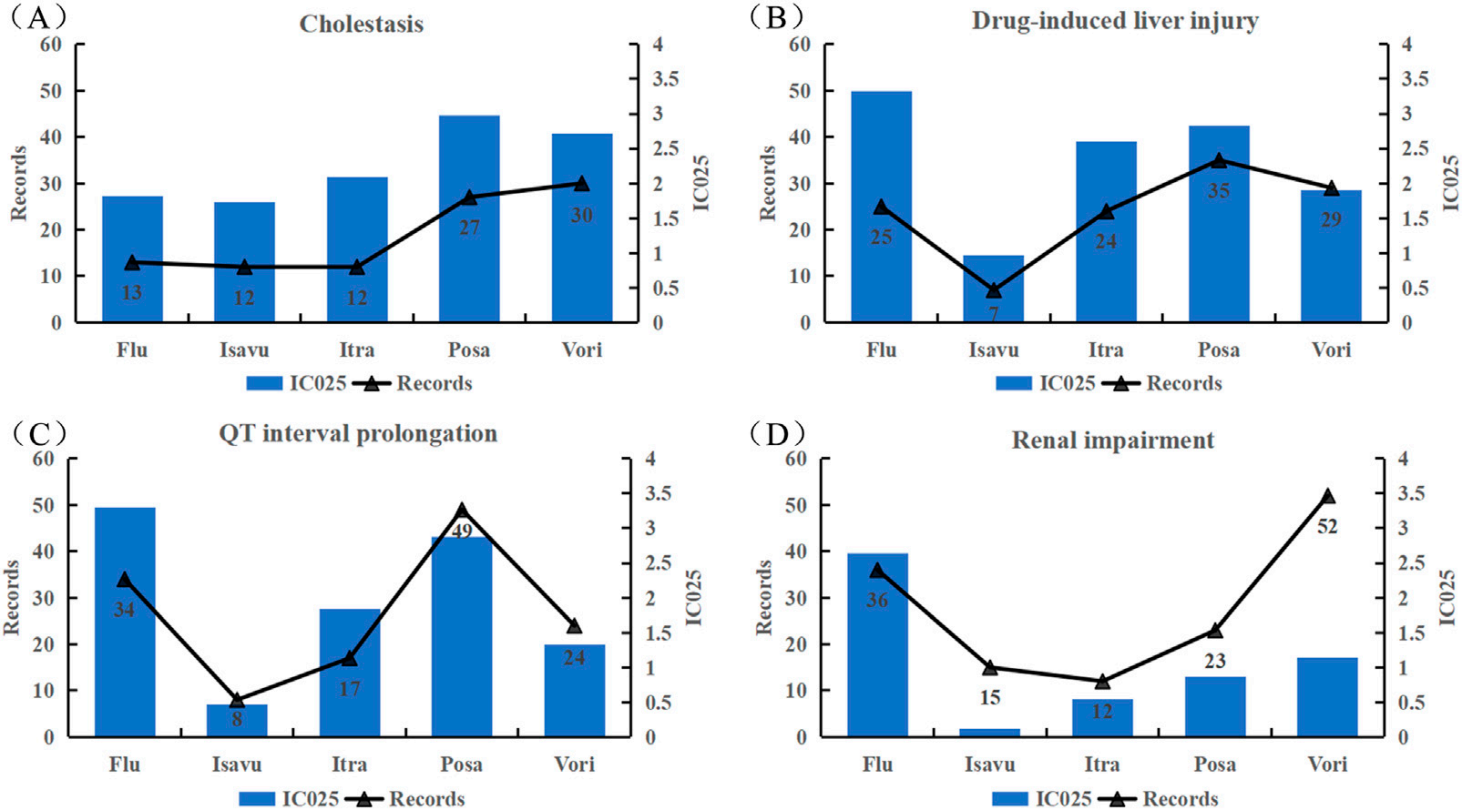
Associations between the four common strong-signal PTs and TAD quantified by IC values. **(A)** Cholestasis; **(B)** Drug-induced liver injury; **(C)** QT interval prolongation; **(D)** boldRenal impairment; Flu: Fluconazole; Isavu: Isavuconazole; Itra: Itraconazole; Posa: Posaconazole; Vori: Voriconazole.

Signal spectrum illustrated that cholestasis, drug-induced liver injury, QT interval prolongation and renal impairment were the most common significant signals for all the TAD. We analyzed their clinical outcomes and the degree of association. Table 5 summarizes the clinical outcomes. A total of 484 clinical outcomes were found (242 other clinical outcomes were not listed), of which severe clinical outcomes accounted for nearly 50%. Further analysis of association between TAD and IMEs (Figure 5) indicated that: cholestasis was strongly associated with posaconazole and voriconazole; drug-induced liver injury was strongly associated with fluconazole and voriconazole; fluconazole and posaconazole were likely to contribute to the development of QT interval prolongation; fluconazole was strongly associated with renal impairment. Isavuconazole showed low intensity in the latter three IMEs (corresponding IC025 = 0.97, 0.47, 0.12).

**TABLE 5:** Common significant signals for serious outcomes in TAD.

Serious adverse event outcome	Significant signals	Records (n, %)
Death	Cholestasis	9 (1.86%)
	Drug-induced liver injury	13 (2.69%)
	QT interval prolongation	12 (2.48%)
	Renal impairment	20 (4.13%)
Life-threatening	Cholestasis	3 (0.62%)
	Drug-induced liver injury	7 (1.45%)
	QT interval prolongation	18 (3.72%)
	Renal impairment	6 (1.24%)
Hospitalization	Cholestasis	47 (9.71%)
	Drug-induced liver injury	35 (7.23%)
	QT interval prolongation	33 (6.82%)
	Renal impairment	31 (6.40%)
Disability	Drug-induced liver injury	1 (0.21%)
	QT interval prolongation	1 (0.21%)
	Renal impairment	3 (0.62%)
Required intervention	Cholestasis	1 (0.21%)
	Drug-induced liver injury	1 (0.21%)
	QT interval prolongation	1 (0.21%)

## 4 Discussion

In this study, the statistical methods of ROR and IC were used to mine the FAERS database for potential adverse effect signals of TAD. Based on different statistical ideas (ROR belongs to the frequency method and IC is based on Bayesian method), the two methods can be validated against each other to reflect the association of target drugs with AEs rapidly and quantitatively. In addition to the investigation of all the TAD-related AEs, IMEs was analyzed, and the important potential adverse reaction signal spectrum was mapped to detect the relevant information intuitively.

### 4.1 Distribution of TAD-related AEs/IMEs at the SOC level

“Endocrine disorders”, “Hepatobiliary disorders”, “Infections and infestations” and “Cardiac disorders” were the four categories that exhibited significant signals in SOC, which is consistent with the observation found in the randomized controlled clinical trials (Neofytos et al., 2010; Uludağ et al., 2013; Kyriakidis et al., 2017; Tantiprawan et al., 2021). TAD can inhibit the activity of cytochrome P450 enzymes, thus induce the hepatotoxicity. By affecting cardiac repolarization, as well as inhibiting hepatic metabolism of other QT prolonging drugs, TADs may induce torsades de pointes (De Ponti et al., 2002).

IMEs included in the instructions were detected in the SOCs mentioned above (e.g., agranulocytosis, cholestasis and inappropriate antidiuretic hormone secretion), which demonstrated the reliable predictability of adverse reaction signal mining based on the Spontaneous Reporting System (SRS). However, there are also serious adverse reactions that are not included in the indication, which require additional attention (e.g., Rhabdomyolysis and Hepatitis fulminant). Our results indicates that before starting treatment, prescribers should pay attention to comorbidity of the patients in the above system and maintain surveillance to ensure the best outcome.

### 4.2 TAD-related IMEs are partially overlapping

Overlapped of TAD-related IMEs can be viewed in the signal spectrum in Online Resource 2. Cholestasis, drug-induced liver injury, QT interval prolonged and renal impairment, torsade de pointes, and erythema multiforme were the common TAD-related signals, either documented in the instructions or demonstrated in the previous studies or clinical trials (Owens, 2004; Yoshikado et al., 2011; Tverdek et al., 2016; Dilokpattanamongkol et al., 2017). When TAD-induced hepatic damage occurs, the secretion and synthesis of bile by hepatocytes is affected. Inhibition of the bile acid salt output pump may cause drug-induced biliary stasis. Accumulated bile acts on the liver, eventually leading to liver damage. Significantly elevated AKP, γ-GGT and TBil in patients taking itraconazole may indicate the suffering from cholestatic liver injury (Yoshikado et al., 2011). A study demonstrated that cholestasis induced by posaconazole and itraconazole was associated with the inhibition of MRP3-mediated phosphatidylcholine secretion (Mahdi et al., 2016). Clinical studies have shown that patients suffer from cholestasis after the use of voriconazole and fluconazole (Bhat et al., 2011; Mihăilă, 2015). These findings are consistent with the signal mined in this study. We found that cholestasis was more likely occurred in patient taking posaconazole and voriconazole, while drug-induced liver injury was associated to a greater extent with fluconazole and voriconazole (Figure 5). TAD can lead to prolonged cardiac repolarization. Despite its low incidence, it has received increasingly widespread attention because of its potentially high risk of causing severe malignant arrhythmias and even sudden death (Shah, 2010). The IME signal of QT interval prolongation associated with TAD was found to be very strong (The mean value of IC025 is 1.96 and the maximum value is 3.3), which is consistent with the results of a network meta-analysis and systematic review (Yang et al., 2021). For intravenous voriconazole, posaconazole and itraconazole, nephrotoxicity is associated with the use of cyclodextrin or sulfobutylether-β-cyclodextrin (Kiser et al., 2015).

Ninety-seven PTs in the IME signal spectrum of TAD were not included in the instructions, mainly distributed in “Endocrine disorders”, “Hepatobiliary disorders” and “Infections and infestations”. All the TAD showed signals of liver damage. Identified unlabeled serious AEs such as pancreatitis acute and hepatitis fulminant can cause serious medical consequences. Clinical application of such drugs for antifungal therapy requires vigilance against adverse reactions in the hepatobiliary system. To reduce the impact of adverse reactions on patients, it is necessary to dynamically monitor the patients’ transaminases, bilirubin, and other indicators before and after TAD administration. When there are signs of abnormal liver function, timely application of hepatoprotective drugs or drugs for the treatment of cholestasis is recommended (Yu et al., 2017).

In this study, isavuconazole not only had less IME signals in single-drug signal mining, but it also had the lowest overall incidence of IME in the combination drug analysis. It can be partly due to the fact that isavuconazole is a relatively newer drug comparing to other TADs and has less FEARS records. On the other hand, it is reported that isavuconazole can be used in smaller doses to obtain better clinical results, (Seifert et al., 2007; Astvad et al., 2017), and therefore the probability of IMEs could be lower in isavuconazole. We also found that the probability of QT interval prolongation and renal impairment with isavuconazole is much lower than that of other TADs. Therefore, isavuconazole can be given priority in clinical use for patients with concomitant related systemic diseases, provided that the antimicrobial spectrum is applicable.

### 4.3 TAD and genetic background

TADs are both CYP substrates and inhibitors. CYP enzymes such as CYP3A4, 3A5, 2C19 and 2C9 are involved in the hepatic metabolism of TADs. Genetic polymorphisms in these enzymes can lead to population-specific variations in drug safety (Amsden and Gubbins, 2017). Currently, studies demonstrate the pharmacogenomics had minimal influences on the safety, tolerability and efficacy of itraconazole, posaconazole and isavuconazole. Pharmacogenomics of CYP2C9 do not appear to effect fluconazole safety and efficacy. Conversely, there are significant pharmacogenomic considerations for voriconazole because it interacts with several polymorphic CYPs, most notably CYP2C19. The wild type (CYP2C19*1A) encodes an enzyme with normal function, whereas other variant alleles encode enzymes with increased (e.g., *17), decreased or absent (e.g., *2-*8) activity. The deletion alleles (e.g., *2 and *3) are more common in Asians, but rare in Caucasians and African-Americans (Hirota et al., 2013; Isvoran et al., 2017). CYP2C19*17 allele with enhanced activity is more prevalent in Europeans and Africans (Moriyama et al., 2017). Researchers determined that voriconazole concentrations were strongly influenced by CYP2C19 polymorphisms (Jin et al., 2016), and 27% of patients with voriconazole trough values greater than 6 mg/L had abnormal liver function (Denning et al., 2002). It seems genetic background cannot explain why more cases were reported in the U.S. (Table 3.) The relationship between genotype and adverse drug reaction is not explored due to the lack of information on patient genotype in the FAERS database.

### 4.4 TAD and fungal breakthrough infections

Multiple unlabeled IMEs associated with fungal infections (e.g., Aspergillus pneumoniae) were also identified in this study, even after AEs of non-TAD antifungal drugs were used as analysis context. Some PTs may contribute to the statistical bias due to their association with the patients’ own underlying diseases. In addition, TADs have a broad spectrum against fungi such as *candida*, aspergillus, mucorales, and cryptococcus. However, each TAD has its specific spectrum, which may lead to fungal breakthrough due to the uncovered strains. Fluconazole, itraconazole and voriconazole have almost no antifungal activity against mucorales, while isavuconazole and posaconazole are potent against Mucorales and aspergillus (Nett and Andes, 2016; Ellsworth and Ostrosky-Zeichner, 2020). Fluconazole has superior activity against cryptococcus and *candida* than other TADs, but without potency against aspergillus (Pahls and Schaffner, 1994). Emergence of triazole-resistant fungal strains can make treatment of fungal disease problematic. There are more than 40 mutated sites of CYP51A protein that cause resistance to TADs. For example, Mutations at the G138 and G448 sites alter the spatial conformation of CYP51A protease, weakening the affinity for azole antifungal drugs (Jenks et al., 2020). In conclusion, this study identified a TAD-related breakthrough fungal infection profile. This should be included in antimicrobial stewardship programs and as an important component. In addition, there may be an urgent need to develop a new class of antifungal drugs in response to the problem of drug resistance.

### 4.5 Limitations and strengths

Our study has several limitations. Firstly, FAERS has inherent flaws as an SRS, such as duplicate records with mixed informative quality. Despite of manual correction and deletion, a small number of cases with duplication may exist. Secondly, although DPA method is commonly used in the ADR excavation, it lacks a gold standard for assessing the significance of suspected adverse reactions. Finally, it is important to note that the monitored significant AE signals represent only potential associations but not causality. Nevertheless, as one of the largest databases of adverse drug events in the world, FAERS remains a very important source in pharmacovigilance research. In the current study, potential IME signals associated with TAD were systematically mined, identified, and visualized by signal spectra, which can provide valuable evidence for further research and clinical use.

## 5 conclusion

We systematically mined TAD-related IMEs for significant signals. We mapped visual signals to comprehensively explore the potential characteristics of TAD-related IMEs. Common IMEs mentioned in the instructions such as cholestasis were detected, and serious IMEs such as hepatitis fulminant that were not included in the prescription label were also identified. Our study found a large proportion of clinically serious outcomes associated with TAD, with cholestasis, drug-induced liver injury, QT interval prolongation and renal impairment causing 50% of all the outcomes. IMEs in isavuconazole were less, showing superiority in drug safety perspectives. All these findings suggest that physicians need to pay more attention to the use of TAD. However, due to the limitations of FAERS, the current study can only reveal a potential increase in the frequency of these IMEs and does not directly prove a causal relationship between the associated adverse reactions and the drugs. Further clinical observation is needed to provide more evidence for the potential AE and IME of TAD.

## Data Availability

The original contributions presented in the study are included in the article/Supplementary Materials, further inquiries can be directed to the corresponding author.

## References

[B1] Amsden J. R. Gubbins P. O. (2017). Pharmacogenomics of triazole antifungal agents: Implications for safety, tolerability and efficacy. Expert Opin. Drug Metab. Toxicol. 13, 1135–1146. 10.1080/17425255.2017.1391213 29022838

[B2] Astvad K. M. T. Hare R. K. Arendrup M. C. (2017). Evaluation of the *in vitro* activity of isavuconazole and comparator voriconazole against 2635 contemporary clinical Candida and Aspergillus isolates. Clin. Microbiol. Infect. 23, 882–887. 10.1016/j.cmi.2017.03.023 28373148

[B3] Benitez L. L. Carver P. L. (2019). Adverse effects associated with long-term administration of azole antifungal agents. Drugs 79, 833–853. 10.1007/s40265-019-01127-8 31093949

[B4] Bhat V. Fojas M. Saslow J. G. Shah S. Sannoh S. Amendolia B. (2011). Twice-weekly fluconazole prophylaxis in premature infants: Association with cholestasis. Pediatr. Int. 53, 475–479. 10.1111/j.1442-200X.2010.03286.x 21040197

[B5] De Ponti F. Poluzzi E. Cavalli A. Recanatini M. Montanaro N. (2002). Safety of non-antiarrhythmic drugs that prolong the QT interval or induce torsade de pointes: An overview. Drug Saf. 25, 263–286. 10.2165/00002018-200225040-00004 11994029

[B6] Denning D. W. Ribaud P. Milpied N. Caillot D. Herbrecht R. Thiel E. (2002). Efficacy and safety of voriconazole in the treatment of acute invasive aspergillosis. Clin. Infect. Dis. 34, 563–571. 10.1086/324620 11807679

[B7] Dilokpattanamongkol P. Panusitthikorn P. Boonprasert R. Chayakulkeeree M. Rotjanapan P. (2017). A case report of intravenous posaconazole in hepatic and renal impairment patient with invasive Aspergillus terreus infection: Safety and role of therapeutic drug monitoring. BMC Pharmacol. Toxicol. 18, 8. 10.1186/s40360-017-0115-z 28143591 PMC5282663

[B8] Ellsworth M. Ostrosky-Zeichner L. (2020). Isavuconazole: Mechanism of action, clinical efficacy, and resistance. J. Fungi. 6, 324. 10.3390/jof6040324 PMC7712939 33260353

[B9] Gomes M. Z. R. Mulanovich V. E. Jiang Y. Lewis R. E. Kontoyiannis D. P. (2014). Incidence density of invasive fungal infections during primary antifungal prophylaxis in newly diagnosed acute myeloid leukemia patients in a tertiary cancer center, 2009 to 2011. Antimicrob. Agents Chemother. 58, 865–873. 10.1128/AAC.01525-13 24277033 PMC3910838

[B10] Gubbins P. O. (2011). Triazole antifungal agents drug-drug interactions involving hepatic cytochrome P450. Expert Opin. Drug Metab. Toxicol. 7, 1411–1429. 10.1517/17425255.2011.627854 21995615

[B11] HHS Food and Drug Administration (2016). International conference on harmonisation; electronic transmission of postmarket individual case safety reports for drugs and biologics, excluding vaccines; availability of Food and drug administration regional implementation specifications for ICH E2B(R3h) reporting to the food and drug administration adverse event reporting system. Fed. Regist. 81, 40890–40891. 27373012

[B12] Hirota T. Eguchi S. Ieiri I. (2013). Impact of genetic polymorphisms in CYP2C9 and CYP2C19 on the pharmacokinetics of clinically used drugs. Drug Metab. Pharmacokinet. 28, 28–37. 10.2133/dmpk.dmpk-12-rv-085 23165865

[B13] Hu Y. Bai Z. Tang Y. Liu R. Zhao B. Gong J. (2020). Fournier gangrene associated with sodium-glucose cotransporter-2 inhibitors: A pharmacovigilance study with data from the U.S. FDA adverse event reporting system. J. Diabetes Res. 2020, 3695101. 10.1155/2020/3695101 32695827 PMC7368210

[B14] Isvoran A. Louet M. Vladoiu D. L. Craciun D. Loriot M.-A. Villoutreix B. O. (2017). Pharmacogenomics of the cytochrome P450 2C family: Impacts of amino acid variations on drug metabolism. Drug Discov. Today 22, 366–376. 10.1016/j.drudis.2016.09.015 27693711

[B15] Jenks J. D. Cornely O. A. Chen S. C.-A. Thompson G. R. Hoenigl M. (2020). Breakthrough invasive fungal infections: Who is at risk? Mycoses 63, 1021–1032. 10.1111/myc.13148 32744334

[B16] Jin H. Wang T. Falcione B. A. Olsen K. M. Chen K. Tang H. (2016). Trough concentration of voriconazole and its relationship with efficacy and safety: A systematic review and meta-analysis. J. Antimicrob. Chemother. 71, 1772–1785. 10.1093/jac/dkw045 26968880 PMC4896404

[B17] Kiser T. H. Fish D. N. Aquilante C. L. Rower J. E. Wempe M. F. MacLaren R. (2015). Evaluation of sulfobutylether-β-cyclodextrin (SBECD) accumulation and voriconazole pharmacokinetics in critically ill patients undergoing continuous renal replacement therapy. Crit. Care 19, 32. 10.1186/s13054-015-0753-8 25645660 PMC4338618

[B18] Kyriakidis I. Tragiannidis A. Munchen S. Groll A. H. (2017). Clinical hepatotoxicity associated with antifungal agents. Expert Opin. Drug Saf. 16, 149–165. 10.1080/14740338.2017.1270264 27927037

[B19] Maertens J. A. Raad I. I. Marr K. A. Patterson T. F. Kontoyiannis D. P. Cornely O. A. (2016). Isavuconazole versus voriconazole for primary treatment of invasive mould disease caused by Aspergillus and other filamentous fungi (SECURE): A phase 3, randomised-controlled, non-inferiority trial. Lancet 387, 760–769. 10.1016/S0140-6736(15)01159-9 26684607

[B20] Mahdi Z. M. Synal-Hermanns U. Yoker A. Locher K. P. Stieger B. (2016). Role of multidrug resistance protein 3 in antifungal-induced cholestasis. Mol. Pharmacol. 90, 23–34. 10.1124/mol.116.103390 27112167

[B21] Mihăilă R.-G. (2015). Voriconazole and the liver. World J. Hepatol. 7, 1828–1833. 10.4254/wjh.v7.i14.1828 26207164 PMC4506940

[B22] Molloy S. F. Bradley J. Karunaharan N. Mputu M. Stone N. Phulusa J. (2018). Effect of oral fluconazole 1200 mg/day on QT interval in African adults with HIV-associated cryptococcal meningitis. AIDS 32, 2259–2261. 10.1097/QAD.0000000000001961 30102652 PMC6150187

[B23] Moriyama B. Obeng A. O. Barbarino J. Penzak S. R. Henning S. A. Scott S. A. (2017). Clinical pharmacogenetics implementation consortium (CPIC) guidelines for CYP2C19 and voriconazole therapy. Clin. Pharmacol. Ther. 102, 45–51. 10.1002/cpt.583 27981572 PMC5474211

[B24] Neofytos D. Avdic E. Magiorakos A.-P. (2010). Clinical safety and tolerability issues in use of triazole derivatives in management of fungal infections. Drug Healthc. Patient Saf. 2, 27–38. 10.2147/dhps.s6321 21701616 PMC3108707

[B25] Nett J. E. Andes D. R. (2016). Antifungal agents: Spectrum of activity, Pharmacology, and clinical indications. Infect. Dis. Clin. North Am. 30, 51–83. 10.1016/j.idc.2015.10.012 26739608

[B26] Norén G. N. Hopstadius J. Bate A. (2013). Shrinkage observed-to-expected ratios for robust and transparent large-scale pattern discovery. Stat. Methods Med. Res. 22, 57–69. 10.1177/0962280211403604 21705438 PMC6331976

[B27] Owens R. C. (2004). QT prolongation with antimicrobial agents: Understanding the significance. Drugs 64, 1091–1124. 10.2165/00003495-200464100-00005 15139788

[B28] Pahls S. Schaffner A. (1994). Aspergillus fumigatus pneumonia in neutropenic patients receiving fluconazole for infection due to Candida species: is amphotericin B combined with fluconazole the appropriate answer? Clin. Infect. Dis. 18, 484–486. 10.1093/clinids/18.3.484 8011849

[B29] Pappas P. G. Kauffman C. A. Andes D. R. Clancy C. J. Marr K. A. Ostrosky-Zeichner L. (2016). Executive summary: Clinical practice guideline for the management of candidiasis: 2016 update by the infectious diseases society of America. Clin. Infect. Dis. 62, 409–417. 10.1093/cid/civ1194 26810419

[B30] Rothman K. J. Lanes S. Sacks S. T. (2004). The reporting odds ratio and its advantages over the proportional reporting ratio. Pharmacoepidemiol. Drug Saf. 13, 519–523. 10.1002/pds.1001 15317031

[B31] Sakaeda T. Tamon A. Kadoyama K. Okuno Y. (2013). Data mining of the public version of the FDA adverse event reporting system. Int. J. Med. Sci. 10, 796–803. 10.7150/ijms.6048 23794943 PMC3689877

[B32] Scholl J. H. G. van Puijenbroek E. P. (2016). The value of time-to-onset in statistical signal detection of adverse drug reactions: A comparison with disproportionality analysis in spontaneous reports from The Netherlands. Pharmacoepidemiol. Drug Saf. 25, 1361–1367. 10.1002/pds.4115 27686554

[B33] Seifert H. Aurbach U. Stefanik D. Cornely O. (2007). *In vitro* activities of isavuconazole and other antifungal agents against Candida bloodstream isolates. Antimicrob. Agents Chemother. 51, 1818–1821. 10.1128/AAC.01217-06 17307977 PMC1855565

[B34] Shah R. R. (2010). Drug-induced QT interval shortening: Potential harbinger of proarrhythmia and regulatory perspectives. Br. J. Pharmacol. 159, 58–69. 10.1111/j.1476-5381.2009.00191.x 19563537 PMC2823352

[B35] Sun Q. Y. Xu J. M. Cao Y. B. Zhang W. N. Wu Q. Y. Zhang D. Z. (2007). Synthesis of novel triazole derivatives as inhibitors of cytochrome P450 14alpha-demethylase (CYP51). Eur. J. Med. Chem. 42, 1226–1233. 10.1016/j.ejmech.2007.01.006 17335940

[B36] Tada K. Maruo K. Isogawa N. Yamaguchi Y. Gosho M. (2020). Borrowing external information to improve Bayesian confidence propagation neural network. Eur. J. Clin. Pharmacol. 76, 1311–1319. 10.1007/s00228-020-02909-w 32488331

[B37] TantiprawanJ.SunthornyothinS.Boonchaya-AnantP.SnabboonT. (2021). Posaconazole-induced pseudohyperaldosteronism. Kaohsiung J. Med. Sci. 37, 253–254. 10.1002/kjm2.12325 33231362

[B38] Tverdek F. P. Kofteridis D. Kontoyiannis D. P. (2016). Antifungal agents and liver toxicity: A complex interaction. Expert Rev. anti. Infect. Ther. 14, 765–776. 10.1080/14787210.2016.1199272 27275514

[B39] Uludağ D. Ozdemir N. Tüysüz G. Eroğlu A. G. Celkan T. (2013). Voriconazole induced bradycardia. Pediatr. Hematol. Oncol. 30, 674–676. 10.3109/08880018.2013.775616 23484777

[B40] van Puijenbroek E. P. Bate A. Leufkens H. G. M. Lindquist M. Orre R. Egberts A. C. G. (2002). A comparison of measures of disproportionality for signal detection in spontaneous reporting systems for adverse drug reactions. Pharmacoepidemiol. Drug Saf. 11, 3–10. 10.1002/pds.668 11998548

[B41] Vogel U. van Stekelenborg J. Dreyfus B. Garg A. Habib M. Hosain R. (2020). Investigating overlap in signals from EVDAS, FAERS, and VigiBase. Drug Saf. 43, 351–362. 10.1007/s40264-019-00899-y 32020559 PMC7105447

[B42] Yang Y. L. Xiang Z. J. Yang J. H. Wang W. J. Xu Z. C. Xiang R. L. (2021). Adverse effects associated with currently commonly used antifungal agents: A network meta-analysis and systematic review. Front. Pharmacol. 12, 697330. 10.3389/fphar.2021.697330 34776941 PMC8585744

[B43] Yoshikado T. Takada T. Yamamoto T. Yamaji H. Ito K. Santa T. (2011). Itraconazole-induced cholestasis: Involvement of the inhibition of bile canalicular phospholipid translocator MDR3/ABCB4. Mol. Pharmacol. 79, 241–250. 10.1124/mol.110.067256 21056966

[B44] Yu Y.-C. Mao Y.-M. Chen C.-W. Chen J.-J. Chen J. Cong W.-M. (2017). CSH guidelines for the diagnosis and treatment of drug-induced liver injury. Hepatol. Int. 11, 221–241. 10.1007/s12072-017-9793-2 28405790 PMC5419998

[B45] Yu Z. Liao X. (2022). Torsade de Pointes/QT prolongation associated with antifungal triazoles: A pharmacovigilance study based on the U.S. FDA adverse event reporting system (FAERS). J. Pharm. Pharm. Sci. 25, 237–243. 10.18433/jpps32867 35790147

[B46] Zhai Y. Ye X. Hu F. Xu J. Guo X. Zhuang Y. (2019). Endocrine toxicity of immune checkpoint inhibitors: A real-world study leveraging US Food and drug administration adverse events reporting system. J. Immunother. Cancer 7, 286. 10.1186/s40425-019-0754-2 31694698 PMC6836403

[B47] Zhou X. Ye X. Guo X. Liu D. Xu J. Hu F. (2021). Safety of SGLT2 inhibitors: A pharmacovigilance study from 2013 to 2021 based on FAERS. Front. Pharmacol. 12, 766125. 10.3389/fphar.2021.766125 34987394 PMC8721280

